# Need for insulin to control gestational diabetes is reflected in the ambulatory arterial stiffness index

**DOI:** 10.1186/1471-2393-13-9

**Published:** 2013-01-16

**Authors:** Henna Kärkkäinen, Tomi Laitinen, Nonna Heiskanen, Heli Saarelainen, Pirjo Valtonen, Tiina Lyyra-Laitinen, Esko Vanninen, Seppo Heinonen

**Affiliations:** 1 Department of Obst/Gyn, Kuopio University Hospital, University of Eastern Finland, P.O.B. 1777, Kuopio FIN-70211, Finland; 2 Department of Clinical Chemistry, Kuopio University Hospital, University of Eastern Finland, Kuopio, Finland; 3 Department of Clinical Physiology and Nuclear Medicine, Kuopio University Hospital, University of Eastern Finland, Kuopio, Finland

## Abstract

**Background:**

The aim was to evaluate the metabolic profile in conjunction with vascular function using the ambulatory arterial stiffness index (AASI) in women with uncomplicated pregnancies and in women with gestational diabetes mellitus (GDM).

**Methods:**

Plasma glucose, lipids, HOMA –IR (homeostasis model assessment of insulin resistance) and AASI, as obtained from 24-hour ambulatory blood pressure monitoring in third trimester pregnancy and at three months postpartum, were measured in three groups of women: controls (N = 32), women with GDM on diet (N = 42) and women with GDM requiring insulin treatment (N = 10).

**Results:**

Women with GDM had poorer glycemic control and higher HOMA-IR during and after pregnancy and their total and LDL (low density lipoprotein) cholesterol levels were significantly higher after pregnancy than in the controls. After delivery, there was an improvement in AASI from 0.26 ± 0.10 to 0.17 ± 0.09 (P = 0.002) in women with GDM on diet, but not in women with GDM receiving insulin whose AASI tended to worsen after delivery from 0.30 ± 0.23 to 0.33 ± 0.09 (NS), then being significantly higher than in the other groups (P = 0.001-0.047).

**Conclusions:**

Women with GDM had more unfavorable lipid profile and higher blood glucose values at three months after delivery, the metabolic profile being worst in women requiring insulin. Interestingly, the metabolic disturbances at three months postpartum were accompanied by a tendency towards arterial stiffness to increase in women requiring insulin.

## Background

Gestational diabetes mellitus (GDM) is defined as glucose intolerance with its onset or first recognition during pregnancy and it occurs in 3–6% of all pregnancies [1]. In Finland, the frequency is even higher, up to 8% [2]. Individuals with GDM exhibit a tendency to develop diabetes in later life [3,4], and it also exhibits an association with pregnancy-induced hypertension [5]. The metabolic syndrome with dyslipidemia as one of its components is more common in women with previous GDM [6,7]. During a GDM pregnancy, the maternal lipids are strong predictors for fetal lipids and abnormal growth [8], with high maternal triglyceride levels leading to fetal macrosomia. When dietary therapy is not successful in maintaining appropriate glucose values in GDM pregnancy, medication is required, traditionally this has been insulin. Usually insulin therapy can be discontinued after delivery. The vascular changes in GDM have been studied in many different ways, mainly at the endothelial level [9]. Paradisi et al. used FMD (flow mediated dilation) to reveal that endothelial dysfunction was present in those pregnancies complicated by GDM [10]. In addition, a history of GDM has been shown to be a risk for endothelial dysfunction [11,12] and increased wall stiffness in the common carotid artery [13], even after the glucose tolerance has normalized.

In non-pregnant subjects arterial stiffness is a strong predictor of risk of suffering cardiovascular events. Many techniques have been used for measuring large artery stiffness, each with their own strengths and limitations [14]. The ambulatory arterial stiffness index (AASI) is a novel index, which is defined as 1 minus the regression slope of the diastolic and systolic blood pressure values in individual subjects being determined from non-invasive 24-hour ambulatory blood pressure recordings [15]. AASI has been shown to correlate with more classic techniques such as pulse wave velocity and augmentation index and it is claimed to be able to detect arterial dysfunction at a younger age than can be achieved with pulse pressure [16]. The normal values of AASI have been proposed to be <0.50 at 20 years and <0.70 at 80 years [15,16]. AASI has been shown to predict cardiovascular deaths and strokes more precisely than classical risk factors even in normotensive subjects [15,17] and to be associated with target organ damage in individuals with arterial hypertension, i.e. left ventricular hypertrophy, carotid artery abnormalities and reduced renal function. [18,19]. Previously we have studied AASI in uncomplicated singleton and twin pregnancies [20].

Blood pressure displays a circadian pattern with a blood pressure reduction (dipping) during the night-time, and this also occurs during normal pregnancy. Frequently, a 10% fall has been used as a cutoff for a normal blood pressure daytime-night-time reduction. In many previous studies, a reduction in the decline in the nocturnal blood pressure has been claimed to be a predictor for cardiovascular risk [21-23] and it also has been shown to be associated with arterial stiffness [24].

The hypothesis for the present study was that women with GDM would display changes in lipids and vascular function both during and after pregnancy. Specifically, this study set out to determine the changes in lipids and in vascular function using AASI in the third trimester of pregnancy and at 3 months postpartum in women with normal singleton pregnancies and in women affected by GDM being treated with or without insulin.

## Methods

We prospectively studied 84 Caucasian childbearing women (52 with gestational diabetes mellitus and 32 with an uncomplicated pregnancy as controls) during the third trimester. A total of 10 women in the GDM group needed insulin to maintain the target glucose values but the others achieved adequate glycemic control through dietary measures. Three months after delivery, we studied 58 of these women (24 from the diet GDM group, 7 from the group requiring insulin, and 27 controls). Twenty-six women had to be excluded, because they did not come to the second visit after delivery.

Women were recruited from the Kuopio University Hospital maternity clinic, where the control subjects were referred initially for clinical check-up visits for a variety of reasons, but after consultation, the course of their pregnancy was considered as normal. Women with GDM were referred to our clinic because they were diagnosed with this metabolic disturbance. Gestational diabetes was determined as an abnormal 75 g oral glucose tolerance test (OGTT) [normal capillary plasma values after 12 h of fasting being lower than 4.8-11.2-9.9 mmol/l (fasting -1h-2h)]. The criteria levels were adopted and modified from a local study [25]. The OGTT was performed on pregnancy weeks 26–28. Plasma glucose levels were determined by the hexokinase method (Konelab 60i Clinical Chemistry Analyzer, Thermo Electron Co, Finland).

Women with at least one value over the limits were included into the GDM group. Of the GDM group, 91.7% had fasting glucose higher than the reference value, 27.7% had the 1-h value and 4.3% had the 2-h value higher than the reference value.

Women with GDM received structured dietary and exercise advice and were taught home blood glucose monitoring by a diabetes specialist nurse. The recommendation was to measure fasting blood glucose in the morning and postprandial glucose levels 1 h after a meal. If there were values over the limits (fasting glucose >6.0 mmol/l and postprandial >7.5mmol/l) despite nutritional advice and exercise, then insulin-therapy was initiated according to our unit’s protocol. Patients were followed up in our specialized antenatal diabetic clinic according to a well-established care protocol.

### Ambulatory blood pressure recordings

Twenty-four-hour ambulatory blood pressure measurements were conducted using an ambulatory blood pressure system (SpaceLabs 90207; SpaceLabs Medical, Inc., Redmond, Washington, USA.) The cuff was placed in the nondominant arm at the brachial level. We programmed the recorders to take blood pressure readings at 15- minute intervals during the daytime and every 30-minutes during the night-time. The duration of night-time was defined individually for each participant according to their normal rhythm. The values obtained from twenty-four-hour ambulatory blood pressure measurements may be regarded as reproducible [26]. AASI was calculated as 1 minus the regression slope diastolic blood pressure values plotted against systolic pressures obtained from individual twenty-four-hour monitoring [15]. The slope was not forced through the origin.

The nocturnal blood pressure fall was defined as the difference between individual daytime and night-time values of systolic and diastolic pressures, respectively. In our study, individuals with nocturnal systolic blood pressure less than 10 mmHg lower than their values daytime were defined as nondippers.

### Assays

Overnight fasting blood samples were acquired for laboratory measurements. Samples were centrifuged at 2000 g for 10 minutes and serum and plasma were separated. All lipid analyses were performed by standard methods with Konelab 60i Clinical Chemistry Analyzer (Thermo Electron Co, Finland). The triglyceride concentration was determined by GPO-PAP enzymatic, photometric assay (Konelab TRIGLYCERIDES kit, Thermo Electron Co, Finland) and the total serum cholesterol concentration was analyzed by an enzymatic, photometric assay (Konelab CHOLESTEROL kit, Thermo Electron Co, Finland). Concentrations of high density lipoprotein (HDL)-cholesterol and low-density lipoprotein (LDL)-cholesterol were determined by a direct, enzymatic, photometric method (Konelab HDL-CHOLESTEROL and Konelab LDL-CHOLESTEROL kits, Thermo Electron Co, Finland). Serum insulin was measured by the electrochemiluminescence immunoassay method (Cobas 6000 analyzer, Hitachi High Technology Co, Tokyo, Japan). The homeostasis model assessment of insulin resistance (HOMA-IR) was used to estimate the levels of insulin resistance with the equation: HOMA-IR= fasting insulin (mU/l × fasting glucose (mmol/l)/22.5 [27].

All statistical calculations were performed with the SPSS for Windows programs (SPSS, Chicago, IL). Statistical significance of difference between the groups were analysed by the *T*-test and One-Way ANOVA. A generalized linear model was used and BMI was used as a covariate. Post hoc analyses were performed, where appropriate. Data are shown as mean ± s.d. A P-value <0.05 was considered statistically significant.

The study was approved by the Ethics Committee of the Kuopio University Hospital, and all participants provided informed consent.

## Results

**Figure 1:**
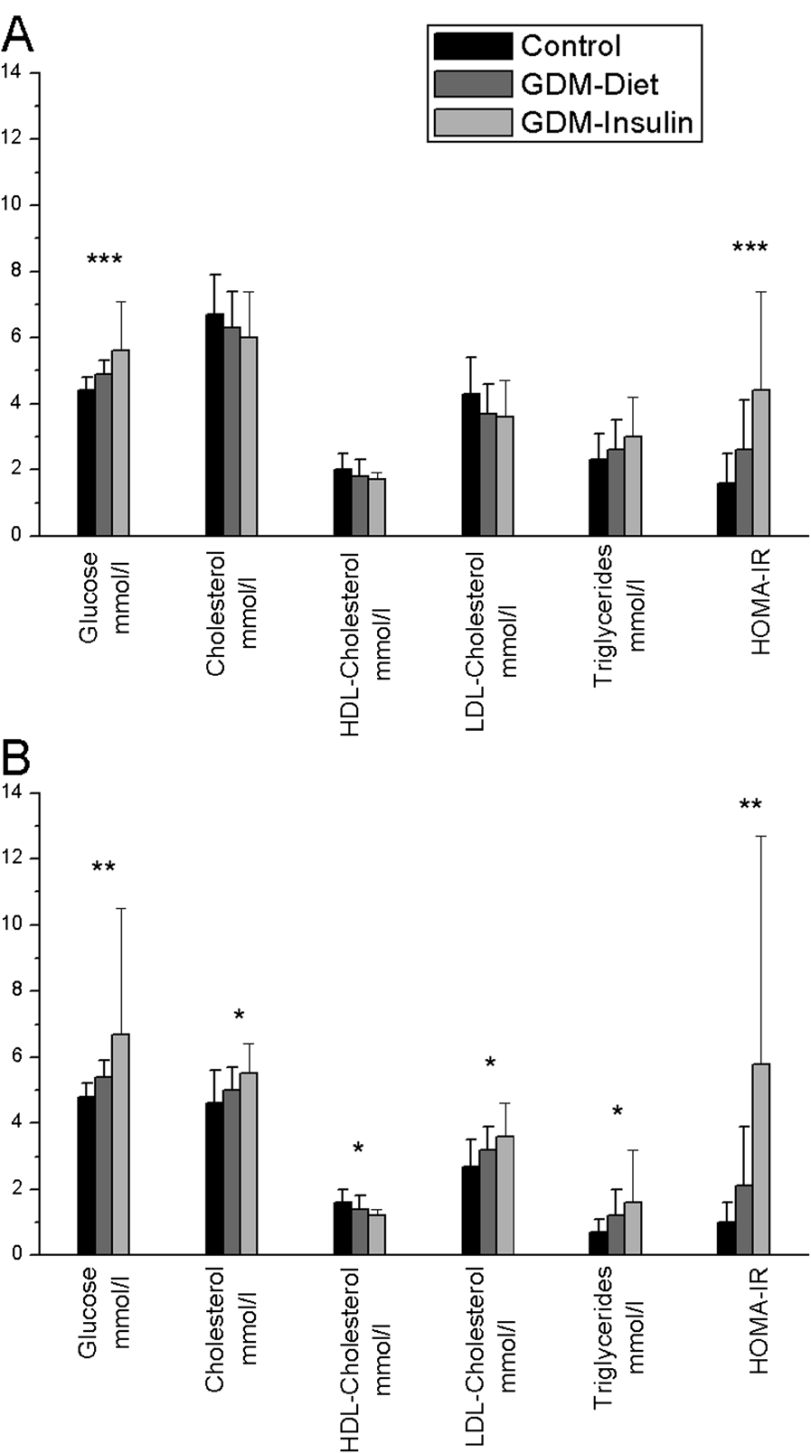
**The differences in metabolic variables between the groups (mean ± standard deviation). A**= during pregnancy, **B**= 3 months after delivery. *= P <0.05, ** =P <0.01, ***=P < 0.001. Comparisons performed using One-Way ANOVA. The numbers of subjects in each group correspond to those in Table 2.

The clinical characteristics of the three study groups are shown in Table 1. There were no differences in the mean maternal age, height or pregnancy weight gain between the groups. The weights of women (P = <0.001) and values of BMI (P = <0.001) before pregnancy in the GDM groups were significantly higher than those of the control group. The mean birth weight in the GDM group was not significantly higher than in controls. During pregnancy, there were marked metabolic differences between GDM and control groups. The mean maternal weight, glucose and HOMA-IR levels were significantly higher in both GDM groups than in the controls (P = 0.000- 0.013) (Figure 1A). There were no significant differences in serum lipids during pregnancy.

**Table 1:** Clinical characteristics of 84 women

**Variable**	**Controls (N = 32)**	**Diet GDM (N = 42)**	**Ins. GDM (N = 10)**
	**(mean ± SD)**		
Age (years)	31.2 ± 4.7	30.5 ± 5.6	32.8 ± 7.6
Height (cm)	166.4 ± 6.8	165.3 ± 5.7	165.4 ± 4.5
Weight before pregnancy (kg)	63.4 ± 7.8†	76.2 ± 16.2*	84.4 ± 21.3*
Weight at birth (kg)	77.1 ± 9.2†	86.8 ± 12.7*	96.9 ± 16.5*
Weight gain (kg)	12.8 ± 3.2	12.9 ± 5.5	11.5 ± 6.3
Weight 3 months after delivery (kg)	63.9 ± 8.3†	78.0 ± 14.8*	81.5 ± 8.3*
Body mass index before pregnancy	22.9 ± 2.9†	27.9 ± 5.5*	30.9 ± 8.3*
Body mass index 3 months after delivery	22.3 ± 2.9†	28.9 ± 5.4*	29.3 ± 2.7*
Gestational age at 1. visit (weeks)	34.1 ± 3.3	33.5 ± 3.1	34.0 ± 2.6
Gestational age at birth (weeks)	39.9 ± 0.92	40.0 ± 1.5	39.1 ± 1.5
Birth weight (grams)	3597 ± 508	3793 ± 480	3744 ± 537
Apgar 1 min	8.78 ± 0.98	8.38 ± 1.82	8.20 ± 1.23
Apgar 5 min	9.00 ± 0.62	8.95 ± 0.67	8.70 ± 0.95

*= significant difference when compared to the control group, †=significant difference when compared to the GDM groups. Significant difference = p <0.05. Comparisons performed using One-Way Anova.

Three months after delivery, there were statistically significant differences between the control group and insulin-using GDM group in all serum lipids and glucose; i.e. there were elevated levels of total cholesterol, LDL, triglycerides and HOMA-IR, whereas the HDL value was lower in GDM insulin group. In women with GDM on diet, the levels of fasting glucose, LDL, triglycerides, and HOMA-IR were significantly higher than in controls (Figure 1B). In the comparison of the situation of the women during their pregnancy and three months after delivery, there were marked changes in all serum lipids (P = 0.000-0.038) in controls and GDM group controlled by diet. In the insulin-receiving GDM group, there was a significant change only in the HDL (P = 0.006), which decreased after pregnancy. After adjustment for BMI, the differences between the groups did not remain statistically significant in terms of total cholesterol, HDL-cholesterol and triglycerides measured after pregnancy. In the control group, the BMI value after delivery was significantly lower than before pregnancy (P = 0.027) but in women with GDM controlled by diet, it tended to be higher (P = 0.092) (Table 1.).

In the comparison between the women controlling GDM by diet and with insulin, there were significant differences in fasting glucose levels during pregnancy (P = 0.011) and HOMA-IR:s during and after pregnancy (P = 0.018, P = 0.044, respectively.) In all of the women treated with insulin, hormone administrations were continued only until the time of delivery.

**Figure 2:**
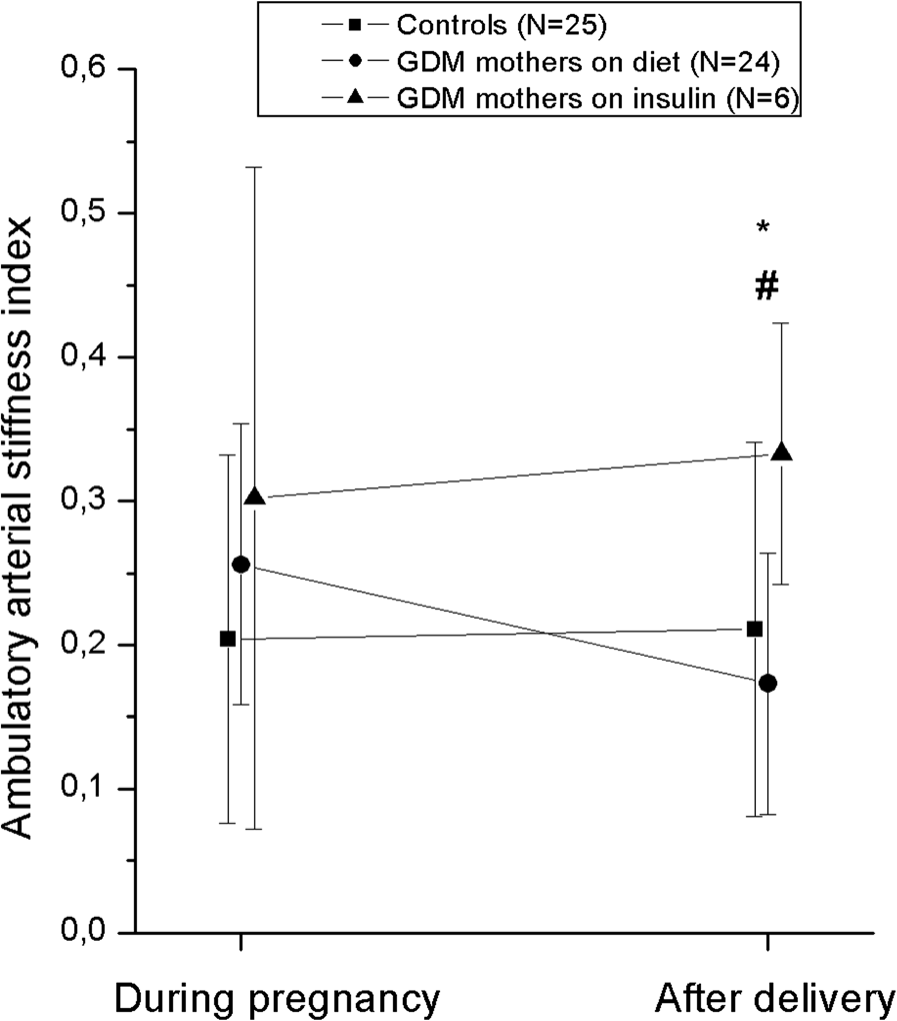
**Ambulatory arterial stiffness index measured during the third trimester and three months after pregnancy in each group. **Only women with two AASI results were included. * = P = 0.047 between the controls and the insulin group, # = P = 0.001 between the GDM groups (Student *t *test was used).

The hemodynamic variables of the three study groups are shown in Table 2. Statistically significant differences between the groups were found both during and after pregnancy in both systolic and diastolic blood pressures, all values being highest in the insulin group, as shown. After adjustment for BMI, the differences did not remain statistically significant in postpartum systolic blood pressures. However, AASI after pregnancy was significantly higher in the insulin group compared to the other groups (Figure 2). AASI was unchanged in the control group during pregnancy and after delivery, but in the GDM group controlled by diet there was a significant improvement in AASI i.e. from 0.26 ± 0.10 to 0.17 ± 0.09 (P = 0.002). In the insulin-using group, the change occurred in the opposite direction, i.e. AASI worsened after delivery from 0.30 ± 0.23 to 0.33 ± 0.09 (Figure 2). The change in AASI (Δ AASI) was defined as AASI after delivery – AASI during pregnancy and the value was negative (−0.08 ± 0.12) in the GDM diet group but positive in the controls (0.01 ± 0.15) and in the GDM group requiring insulin (0.03 ± 0.23). The difference in Δ AASI was significant only between controls and women with GDM on diet (P = 0.021).

**Table 2:** Ambulatory measurements

	**Controls**		**GDM and diet**		**GDM and insulin**	
**Variable**	**1.visit**	**2.visit**	**1.visit**	**2.visit**	**1.visit**	**2.visit**
**mean ± SD**	**(N = 32)**	**(N = 27)**	**(N = 42)**	**(N = 25)**	**(N = 10)**	**(N = 6)**
Systolic BP office (mmHg	109.1 ± 7.5	103.5 ± 7.8 ♦	112.7 ± 10.4	111.9 ± 21.8	116.5 ± 15.9*	115.5 ± 12.1*
Diastolic BP office (mmHg)	68.9 ± 6.3	70.1 ± 5.7	70.1 ± 9.0	71.8 ± 8.7	75.0 ± 7.8*	79.8 ± 9.4*
Systolic BP whole 24 h (mmHg)	114.5 ± 7.0	112.0 ± 7.4	115.2 ± 9.0	113.8 ± 10.5	123.1±13.3†*	120.3 ± 6.0*
Diastolic BP whole 24 h (mmHg)	71.0 ± 5.9	71.7 ± 6.3	68.8 ± 7.4	69.8 ± 8.8	74.3 ± 8.4†	77.5 ± 6.6
Heart Rate 24 h (bpm)	81.8 ± 9.1	69.0 ± 8.6 ♦	85.5 ± 10.6	72.9 ± 9.2♦	80.6 ± 6.6	75.0 ± 6.8♦
Systolic BP daytime (mmHg)	117.7 ± 7.0	115.2 ± 7.4	119.3 ± 9.4	117.7 ± 10.5	125.8 ± 13.2*	123.8 ± 8.5*
Diastolic BP daytime (mmHg)	74.8 ± 5.7	75.3 ± 5.9	73.2 ± 8.0	73.9 ± 8.5	77.4 ± 8.2	81.0 ± 8.7
Heart rate daytime (bpm)	85.4 ± 9.6	73.5 ± 9.2 ♦	89.4 ± 11.0	77.2 ± 9.2♦	94.1 ± 6.9	78.8 ± 7.5♦
Systolic BP night (mmHg)	107.2 ± 8.0	105.7 ± 8.6	106.7 ± 9.3	106.1 ± 11.9	116.7± 15.0†*	115.7 ± 4.4*
Diastolic BP night (mmHg)	62.3 ± 7.2	64.7 ± 7.6 ♦	59.6 ± 7.3	61.0 ± 10.0	67.0 ± 9.5†	72.3 ± 5.3 †*
Heart rate night (bpm)	73.3 ± 9.0	59.4 ± 9.0 ♦	76.7 ± 10.5	63.5 ± 9.8♦	72.9 ± 7.8	67.0 ± 9.4
Nocturnal syst. dipping (mmHg)	10.5 ± 4.3	9.4 ± 5.3	12.6 ± 5.7	11.8 ± 5.8	9.1 ± 5.3	8.2 ± 5.6
Nocturnal diast. dipping (mmHg)	12.6 ± 3.8	10.6 ± 4.2 ♦	13.6 ± 4.7	12.8 ± 5.0	10.4 ± 5.9	8.7 ± 3.9
AASI	0.22 ± 0.13	0.21 ± 0.13	0.23 ± 0.12	0.17 ± 0.09♦	0.29 ± 0.21	0.33 ± 0.09 †*

1. visit, during pregnancy, 2. Visit, three months after delivery. *BP*, blood pressure, *AASI*, ambulatory arterial stiffness index, *bpm*, beats per minute. *, significant difference when compared to the control group, †, significant difference when compared to the GDM diet group, ♦, significant difference when compared to 1.visit. Significant difference = p <0.05.

In all three groups, the heart rates were significantly higher during pregnancy than three months later (P ≤0.000).

The factors correlating with AASI during pregnancy and AASI after delivery are shown in Table 3. AASI during pregnancy correlated positively with systolic blood pressures and night-time blood pressures during pregnancy, woman’s age and with body mass index before pregnancy. A negative correlation was found between AASI during pregnancy and nocturnal diastolic dipping. There was no correlation between AASI values and 2-hour glucose test measurement results. AASI values measured 3 months after delivery correlated positively with values of systolic and diastolic blood pressure during the whole 24 hours, but the most significant association was recorded with night-time blood pressures (P = 0.001-0.003). It also correlated negatively with nocturnal systolic dipping after delivery and diastolic dipping during pregnancy.

**Table 3:** Significant correlations between selected variables and AASI during pregnancy and after delivery

	**AASI During pregnancy (N=84)**	**AASI After delivery (N=58)**
Systolic BP whole 24 h during pregnancy	r = 0.247*	r = 0.212
Systolic BP night-time during pregnancy	r = 0.286**	r = 0.244
Diastolic BP night-time during pregnancy	r = 0.215*	r = 0.204
Systolic BP whole 24h 3 months after delivery	r = 0.225	r = 0.341*
Diastolic BP whole 24h 3 months after delivery	r = 0.032	r = 0.280*
Systolic BP night-time 3 months after delivery	r = 0.227	r = 0.410**
Diastolic BP night-time 3 months after delivery	r = 0.045	r = 0.395**
BMI before pregnancy	r = 0.248*	r = 0.078
Woman’s age	r = 0.245*	r = 0.246
Nocturnal diastolic dipping during pregnancy	r = -0.386**	r = -0.317*
Nocturnal systolic dipping 3 months after delivery	r = -0.077	r = -0.344*

*AASI*, ambulatory arterial stiffness index, *BP*, blood pressure, *BMI*, body mass index. Significant difference =* p < 0.05, ** p = < 0.01.

## Discussion

It has been previously shown that up to one third of women with a history of GDM develop the metabolic syndrome in a period of approximately ten years [6,7]. The present study reveals that a number of metabolic risks such an unfavourable lipid profile, HOMA-IR and higher blood pressure were clustering at three months postpartum in women with GDM and that in women with GDM requiring insulin, the unfavourable metabolic profile was accompanied by significantly higher AASI than in the control women and in women with GDM controlled by nutritional therapy. Overall, at least three interesting conclusions can be drawn from this study. First, in women with GDM controlled by diet, the subtle increase in arterial stiffness observed during pregnancy was found to be totally reversible, although in the postpartum period they did not adhere to the prudent diet to which they were committed during pregnancy. This was reflected by the fact that the lipid profiles during pregnancy, but not postpartum, were similar or even somewhat better in the GDM than in the control group and accordingly the weight gain during, but not after pregnancy, was similar in both groups. Second, those women with GDM receiving insulin appeared to develop more severe dyslipidemia and exhibited an increasing trend in arterial stiffness after delivery, indicating that the metabolic disease in a stepwise manner was accompanied by notable changes in a vascular function and suggesting that there really was a dose and response type of relationship. Savvidou et al.[28] have shown that pregnancies complicated by insulin resistance are associated with systemic maternal arterial stiffness increasing progressively from controls to gestational to type 2 diabetics which our results now confirm. The study of Davenport et al. [29] indicated that the vascular function of women in the early postpartum period is directly influenced by the development of GDM during pregnancy and by the persistence of clinical and/or subclinical hyperglycemia after delivery, a phenomenon observed also in our study.

AASI during pregnancy correlated positively with the mother’s age and the correlation with age and AASI after delivery was also almost statistically significant. These findings are in line with the results of previous studies [16]. Pre-pregnancy BMI correlated also with AASI during pregnancy. Accordingly, the positive correlations with the night-time blood pressure values and the observed negative correlation with nocturnal diastolic dipping are phenomena that have been detected also in previous studies, confirming the validity of the present results [30].

A reduction in the decline in the nocturnal blood pressure is claimed to be a predictor for cardiovascular risk [21-23] and it also has been shown to be associated with arterial stiffness [24]. This was also seen within the groups in the present study, even though the differences between the groups were not statistically significant.

The mechanisms behind the present results remain speculative, but they may be related to fundamentally different metabolism in women with metabolic risks. In the women with GDM, those with the lowest beta-cell function will require insulin in pregnancy [31] and the effect of providing insulin may have had some impact on the results i.e. insulin is known to possess vasodilatory properties and it may thus mask the effect of impaired glucose tolerance [32]. On the other hand, the second half of pregnancy is known to be characterized by acquired insulin resistance that improves immediately after pregnancy. Furthermore, there is also a link between fat mass, lactation, sex steroids and glucose metabolism. We do not have any data on how many of the mothers in our study were breast-feeding, but three months after delivery, the vast majority, up to 78%, of mothers is known to breast-feed in Finland [33]. Lactation is an oestrogen-deficient state, predisposing women to abdominal and visceral fat accumulation. The effect of oestrogen may be reflected in eating or activity behaviours and thus on mechanisms that have a direct effect also on glucose metabolism. Oestrogen treatment in mice is known to increase lipolysis in abdominal fat cells and to modulate leptin responsiveness in several ways. [34]. Oestrogen deficiency in female animals is also known to be associated with hyperphagia and increased body weight, the effects probably being mediated by neuropeptide Y, agouti-related peptide, proopiomelancortin and ghrelin [34,35]. However, little is known about the effects of lactation on vascular function, although the metabolic effects seen in the present study were clear-cut across the full spectrum of gestational glucose intolerance.

Impaired glucose tolerance and hypertensive problems during pregnancy have been considered to be a potential window into a woman’s future [36] revealing tendencies about whether she will suffer cardiovascular problems such as hypertension or diabetes mellitus later in life. In our study, there was transient stiffening in the arteries during pregnancy with a restoration of elasticity after delivery in the group of GDM women who were being controlled by nutritional treatment but not in the insulin requiring group, despite the fact that they adhered to a strict diet. Using the previously established cut-offs [15,16], the AASI values were within the normal age-related limits in both groups and therefore, the clinical significance of the observed AASI changes will require further study. Overall, this may have true clinical significance, as it is believed that AASI predicts cardiovascular complications, even in normotensive subjects.

One weakness of our study was the rather small number of participants and in addition there quite many dropped out. One obvious reason for the considerable number of drop-outs was the presence of the newborn baby, who occupied all the mother’s attention. The optimal time for recording AASI values during pregnancy is not known. On the other hand, there is evidence that arterial elastic properties may worsen during the third trimester in normal pregnancies [37,38], suggesting that AASI measurements would have been different if they had been conducted earlier in the pregnancy. In the current setting, third-trimester pregnant women were enrolled since pregnancy specific changes, such as GDM, had become manifested and were being diagnosed by that time. Accordingly, the normality of the controls could not have been verified earlier than near term, since many obstetric complications only appear in the third trimester. Otherwise, the subjects of the present study were young and were considered to be healthy before and after their pregnancy. The cut-off values for GDM diagnostics in OGTT in Finland have changed since our study, being now 5.3 -10-8.6 mmol/l, and there is the possibility that the results would have been changed slightly if the updated cut-off values had been used.

## Conclusions

In conclusion, GDM women adhering to a strict diet or those receiving insulin therapy, displayed signs of increased arterial stiffness during GDM pregnancy. The observed increase in arterial stiffness was more pronounced in those women requiring insulin, as expected on the basis of the severity of the metabolic disturbance. In women whose GDM was being controlled by dietary measures, the increase in arterial stiffness was reversible whereas in women requiring insulin therapy the observed increase in arterial stiffness still appeared to be present three months after delivery. Nevertheless, the AASI values were within the normal limits in all groups studied and therefore, as yet there are no clinical implications. Nonetheless, it is recognized that those pregnant women who require insulin to maintain the target glucose values are at a true risk of suffering type II diabetes and experiencing cardiovascular complications later in life. AASI is a quite novel, simple and inexpensive method for monitoring arterial stiffness and it may help to identify women at high risk in a timely manner.

## Abbreviations

AASI: Ambulatory arterial stiffness index; GDM: Gestational diabetes mellitus; FMD: Flow mediated dilation; BP: Blood pressure; HOMA-IR: Homeostasis model assessment of insulin resistance; LDL: Low density lipoprotein; HDL: High density lipoprotein; BMI: Body mass index; OGTT: oral glucose tolerance test.

## 

This work was supported by the Kuopio University hospital (EVO-grants 5302419 and 5031316).

## Competing interests

No competing financial interests exist.

## Author’s contributions

All authors participated in concepting and designing the study, HK performed the analysis and interpreted them with TL and SH HK TL and SH participated in drafting the manuscript. All authors have revised the manuscript critically for important intellectual content and read and approved the final manuscript.

## Pre-publication history

The pre-publication history for this paper can be accessed here:

http://www.biomedcentral.com/1471-2393/13/9/prepub
